# Retained self-inserted foreign body into the urethra associated with sequela urethral stenosis: a case report

**DOI:** 10.1186/1752-1947-8-244

**Published:** 2014-07-05

**Authors:** Driss Amiroune, Ahmed Amine Bouchikhi, Fayez Adawi

**Affiliations:** 1 University Hospital Hassan II, Sidi Hrazem Road, 30000 Fez, Morocco; 2 Chambery Hospital, Place Lucien Biset, 73000 Chambery, France

## Abstract

**Introduction:**

Self-insertion of foreign bodies into the urethra represents a heterogeneous group of cases concerning a wide variety of objects and involving multiple procedures and surgical techniques.

**Case presentation:**

We report a case of self-insertion of an electric cable into the urethra by a 36-year-old Caucasian man for erotic reasons. The patient, who has an ambiguous history of self-inflicted penile strangulation in childhood and self-insertion of foreign bodies into the urethra in recent years but no psychiatric history, presented to the emergency department to remove the object introduced one week previously. He was - strangely - asymptomatic and presented neither dysuria nor urinary incontinence or hematuria. A physical examination revealed a penile scar corresponding to the strangulation and a palpable hard, thin mass in the perineal urethra. The biologic findings were normal. Plain film of the urinary tract showed a hollow tubular object, whose size and shape corresponded to those of the urethra. Rigid cystoscopy was performed, which revealed urethral stricture at the projection of the scar. Laborious urethrotomy was performed before reaching the 25cm long cable, which was found in the urethra and removed with difficulty due to stenosis.

**Conclusions:**

We encountered a particular case combining a self-introduced foreign body in the urethra and the sequelae of such manipulations, which is urethral stricture. We succeeded in treating both by endoscopy, which is not always possible in this situation.

## Introduction

Although uncommon, the discovery of a foreign body in the urethra is an event that every physician, especially urologists, may be confronted with during his medical practice. This is a phenomenon described in the early medical literature but is still not well understood [1]. The reasons are often erotic but may be psychotic. The foreign bodies are diverse and their extraction involves, whenever possible, improvised maneuvers or endoscopy to avoid as far as possible recourse to surgery, which sometimes is inevitable. The case we present is interesting in that it deals with the introduction of a foreign body into the urethra together with one of its long-term complications, that is, urethral stricture.

## Case presentation

**Figure 1:**
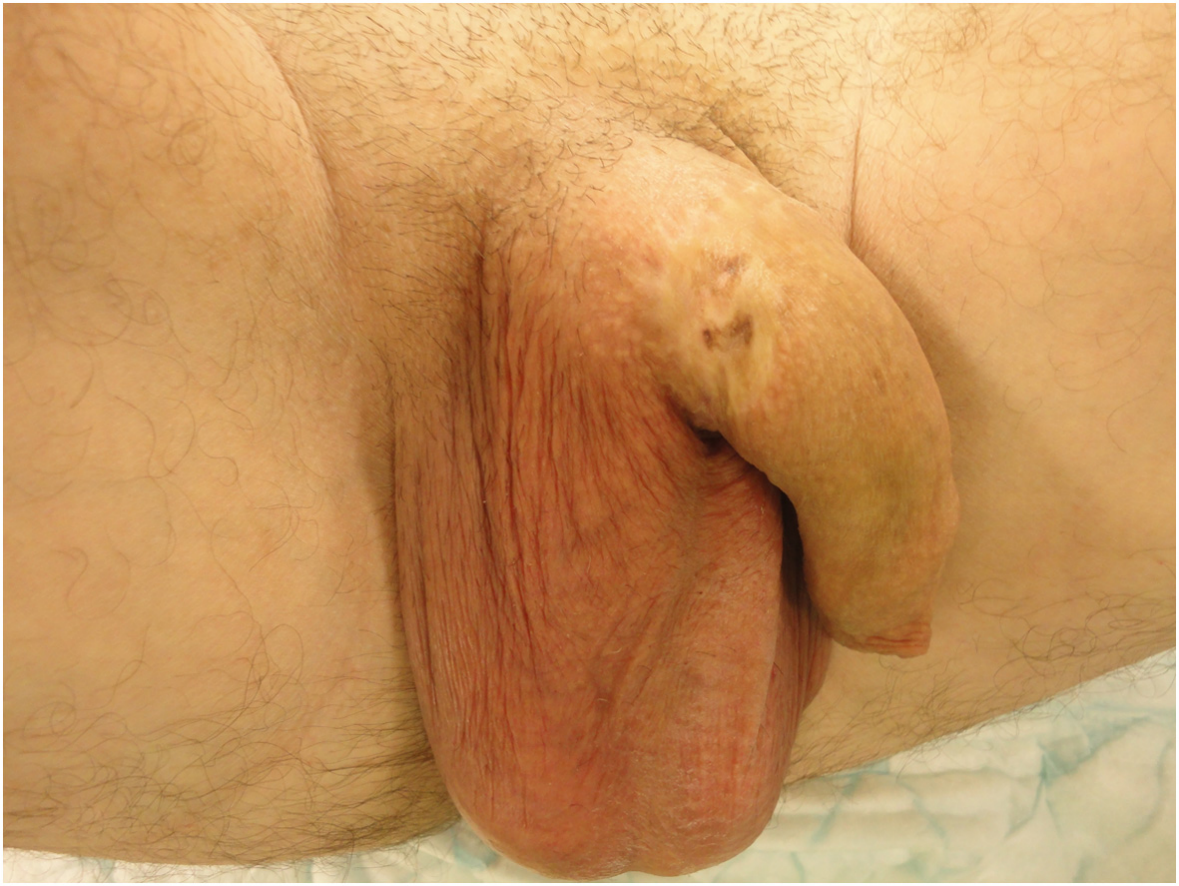
The photo shows the scar resulting from self-inflicted penile strangulation.

We present the case of a 36-year-old Caucasian man with a history of self-inflicted penile strangulation by a ring in childhood. He said that he committed this act at the age of 10 years to ‘play’ but the exact circumstances of this incident remain unclear. Our patient had begun to self-stimulate by introducing pieces of electrical cable into the urethra a few years before the current episode where the cable was completely retained in the urethra. Contrary to what might have been expected, he remained asymptomatic and presented with neither dysuria nor hematuria nor urinary incontinence. A physical examination on admission found an emotionally stable patient with no sign of neurosis or psychosis. An examination of the external genital organs found a scar at the base of the penis corresponding to the history of self-inflicted penile strangulation and a hard mass in the perineal urethra corresponding to the cable (Figure 1). No leakage of urine was found.

**Figure 2:**
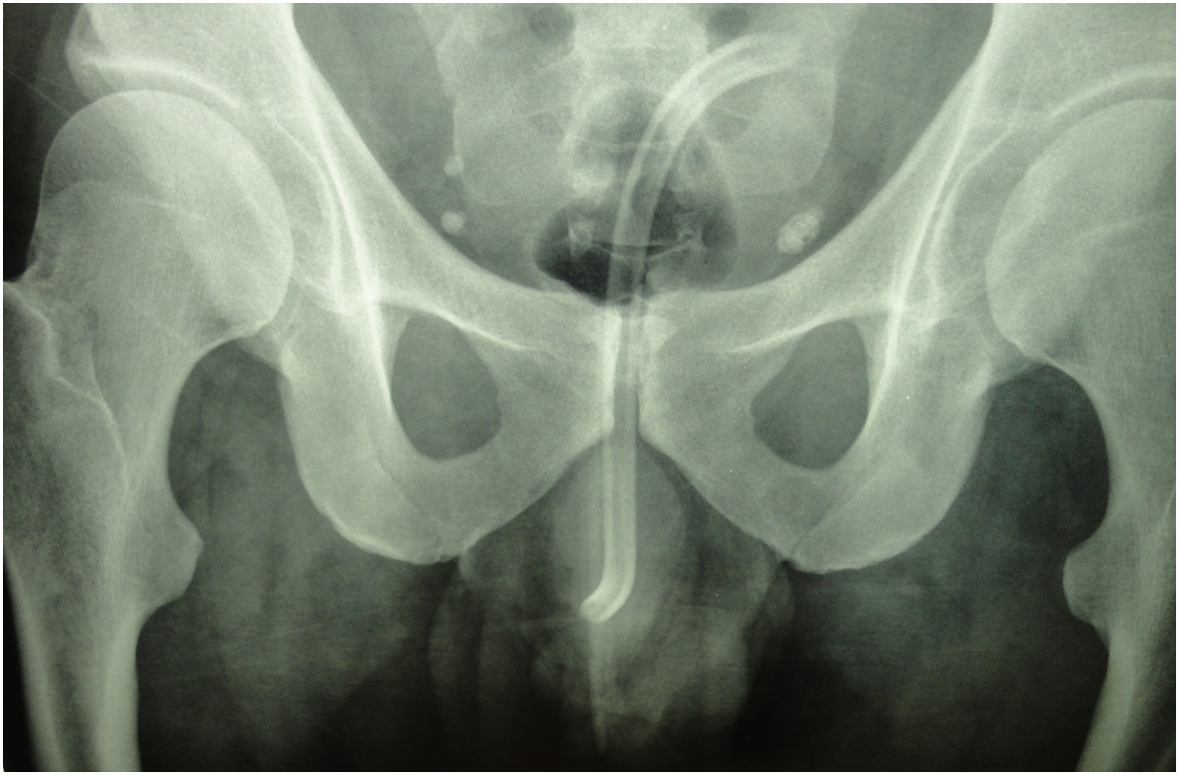
Plain film of the urinary tract showing a hollow tubular object corresponding to a cable in the urethra.

The biologic findings were normal.Plain film of the urinary tract showed a hollow tubular object measuring the size of the urethra and reproducing its shape up to the urinary bladder (Figure 2).

**Figure 3:**
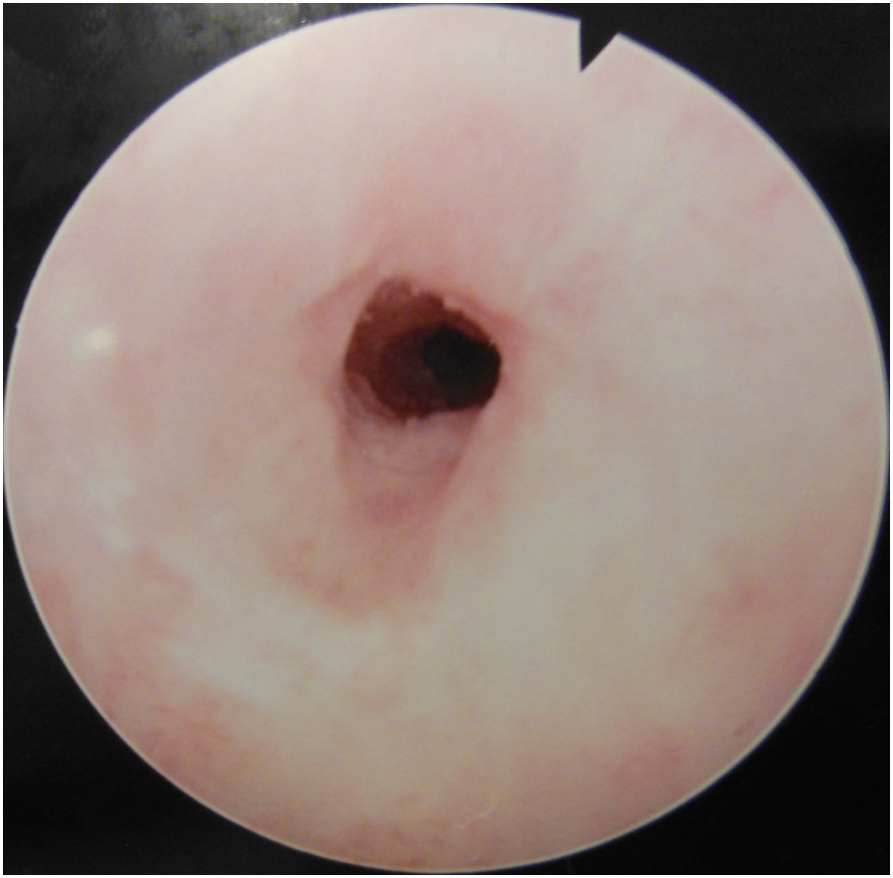
Urethroscopy image of the tight urethral stenosis.

**Figure 4:**
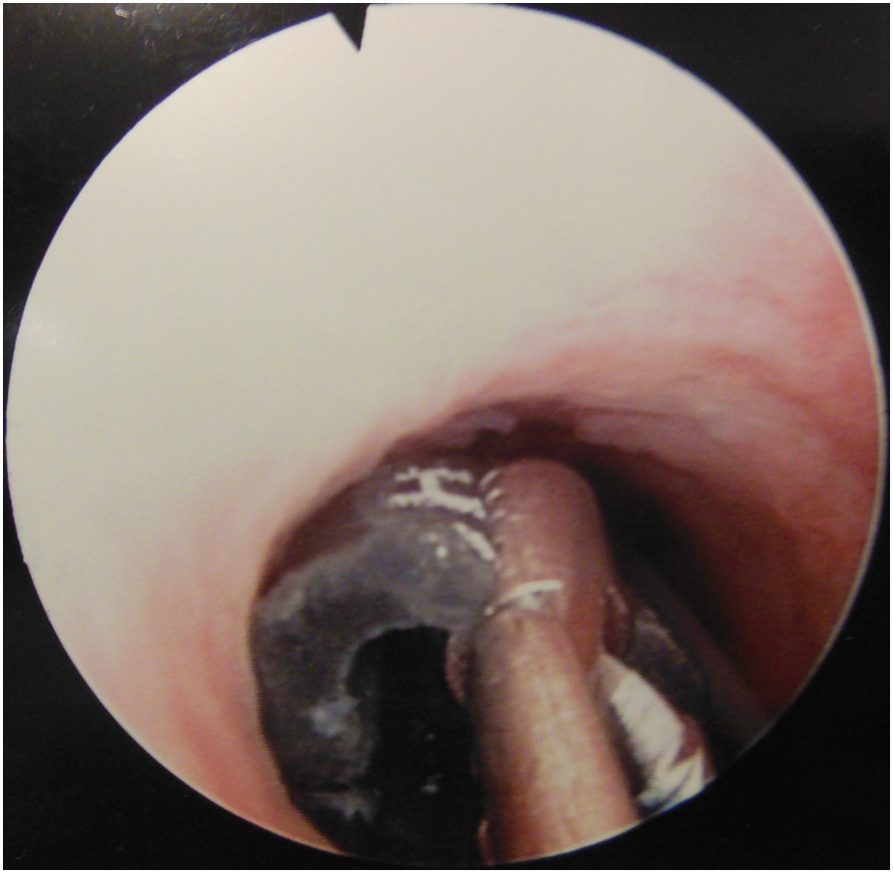
The electric cable in the urethra.

**Figure 5:**
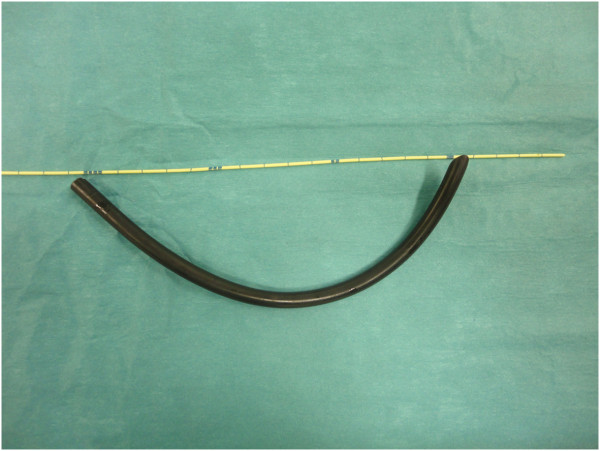
The electric cable as extracted from the urethra.

**Figure 6:**
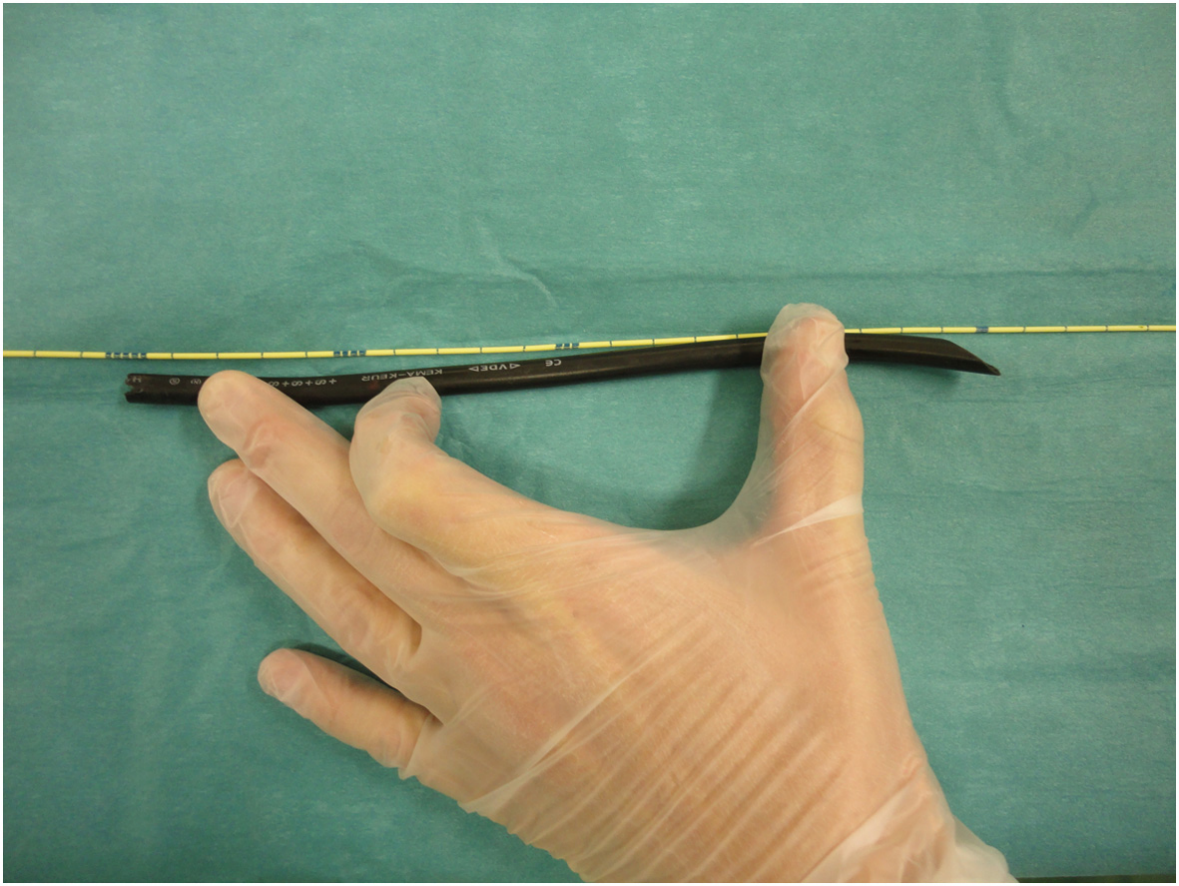
The photo shows the actual length of the cable, which measured 25cm.

The radiopaque image corresponded to an electrical cable that extended from the urethra into the bladder. We opted for endoscopic treatment because the consultation delay was relatively short and since the hollow shape of the cable might allow its capture.During urethroscopy, we were surprised by the presence of a urethral stricture tight enough to block the progression of the cystoscope (Figure 3), but decided to go through with the procedure by performing urethrotomy on the urethral stricture. Urethrotomy was laborious in the face of very fibrotic tissue that was very hard to cut. Finally, we were able to cross the stenosis and reach the extremity of the cable (Figure 4) but its extraction was still hampered by the freshly incised stenosis. After several attempts, we managed to remove the cable, which measured 25cm long and was about 1cm in diameter, without requiring open surgery (Figure 5 and Figure 6).

The procedure ended by placing a bladder catheter inserted over a guide that had been introduced at the beginning of the operation. Our patient was discharged from the hospital after two days and the catheter was removed after 10 days. He did not show any infectious complications.

## Discussion

Many cases reporting self-insertion of foreign bodies in the urethra are described in the literature [2], they affect both sexes, with a male predominance, and all age groups [3]. The foreign body can take any form and be made of any material, it can be synthetic or biological (pencil, cable, pin, toothbrush, squirrel tail…) and sometimes unusual, as in the case of inserting a decapitated snake [3].

Patients with a retained foreign body in the urethra may delay to consult depending on the discomfort felt. Consultation is often motivated by signs such as dysuria, pain or hematuria but the diagnosis can be made fortuitously after many years with no troublesome symptoms [4]. The consequences of this act can sometimes be grave when infectious complications occur such as Fournier’s gangrene [5].

Unlike some other self-performed practices on the penis, such as strangulation or self-amputation, introduction of foreign bodies into the urethra is not necessarily associated with psychiatric disorders or drug abuse although such circumstances are described in the literature. Patients, when they do not deny it, often claim to have done this to provide pleasure, play and in some cases to enlarge their penis or improve urination [1,3,6]. A high frequency of this phenomenon exists among patients with genital piercing [1].

Diagnosis is often easy based on the consultation, a physical examination that may reveal a hard urethral mass, as well as radiography of the urinary tract. When the foreign body is not radiopaque, a pelvic scan or cystoscopy may be necessary [3]. Many means can be used to extract a retained self-introduced foreign body from the urethra. They may be grouped into three categories: nonoperative means (using a basket or forceps) that can challenge the creativity and imagination of the urologist (and, in some cases, the radiologist), endoscopy as in our case, or surgery if necessary, which is a far from rare request [7-9].

The first two options are preferred as much as possible; surgery should remain a last resort and consist of urethrotomy or cystotomy after pushing the object into the bladder. The shape of the foreign body, its consistency and its location, as well as the time of the consultation determines the extraction method [3].

Long-term sequelae of self-inserted foreign bodies into the urethra are represented by urethral stricture, urethral diverticulum and erectile dysfunction [3,9], but it is not easy to relate them to their predisposing cause in practice because patients do not report their habits during the consultation.

In this case, we were confronted with stenosis of the urethra that could have caused the failure of endoscopic retrieval of the foreign body. The stenosis was located on the projection of the old penile strangulation scar so we could not determine of which practice it was a consequence, but it is likely that the two manipulations had contributed to its formation. In any case, our case confirms the long-term danger of such urethral automanipulations because no other factor was found to explain the stenosis in this young patient of 36 years.

## Conclusions

Retained self-inserted foreign body in the urethra is uncommon in clinical practice despite the numerous cases described throughout history. The nature of the inserted object determines the procedure that should be performed for extraction, which should be as minimally invasive as possible. The reported case confirms that urethral stricture is a late sequela of self-manipulation of the urethra in a population that tends to hide this detail in consultation so that this link is rarely clearly made.

## Consent

Written informed consent was obtained from the patient for publication of this case report and any accompanying images. A copy of the written consent is available for review by the Editor-in-Chief of this journal.

## Competing interests

The authors declare that they have no competing interests.

## Authors’ contributions

DA analyzed and interpreted the patient data, did the literature review, participated in the treatment and was the major contributor to the writing of the manuscript. AAB helped write the manuscript, and drafted the manuscript. FA established the treatment and gave final approval of the version to be published. All authors read and approved the final manuscript.
